# List of Accidents Admitted into the Pennsylvania Hospital, from February 14th to March 8th, 1838

**Published:** 1838-03-14

**Authors:** 


*List of Accidents admitted into the Pennsylvania Hospital, from February 14th to March 8th,* 1838.

One extensive burn of the back, treated with poultice and simple cerate; one wouud of the face; one dislocation of the humerus into the axilla, reduced in half a minute, by Dr. Wallace, with the heel in the axilla, and, in addition to the usual extension, the head of the bone being drawn *outwards*, by a towel, passed under it; one contusion of the thigh; one case of poisoning from f℥vi laudanum, on an empty stomach,—the stomach tube was applied two hours and a half afterwards, V. S. ad f℥xx, practised, under which the pulse rose, and the man being comatose, flagellations were resorted to successfully, to rouse him. One severe wound, of the foot, from a broadaxe, dividing the metarsal bones of the great and second toes, and opening the anterior tibial artery and plantar arch. Six ligatures were applied, the edges of the wound drawn together, and the limb elevated. The injury was inflicted 12 hours previous to the man's admission, and it had been dressed merely with a tight bandage. One fracture of the radius, just above the wrist joint, which was brought in, dressed with splints, from the fingers to just above the fracture. In consequence of the severe pressure from the swelling which followed this dressing, sloughing occurred over the wrist joint, which complicated the case materially.

The burn of the back, reported in No. 4, has been discharged cured by Goulard's cerate, in three weeks. The incised wound, reported in same number, cured in four weeks.

